# Targeting Neuroinflammation and Oxidative Stress to Slow Neurodegeneration in the Visual System

**DOI:** 10.3390/jcm15093254

**Published:** 2026-04-24

**Authors:** Nara Shakaki, Minzhong Yu

**Affiliations:** 1 College of Medicine, Northeast Ohio Medical University, Rootstown, OH 44272, USA; nshakaki@neomed.edu; 2 Department of Ophthalmology and Visual Sciences, University Hospitals Eye Institute, Case Western Reserve University, Cleveland, OH 44106, USA; 3 Cole Eye Institute, Cleveland Clinic Foundation, Cleveland, OH 44106, USA; 4 Department of Ophthalmology, Cleveland Clinic Lerner College of Medicine of Case Western Reserve University, Cleveland, OH 44195, USA

**Keywords:** glaucoma, age-related macular degeneration, diabetic retinopathy, Alzheimer’s disease, neuroinflammation, oxidative stress

## Abstract

Purpose: Neuroinflammation and oxidative stress are increasingly recognized as central, interconnected drivers of neurodegeneration in the visual system. This review examines the pathogenic mechanisms shared across glaucoma, age-related macular degeneration (AMD), diabetic retinopathy (DR), and Alzheimer’s disease (AD), and evaluates the therapeutic rationale for targeting both pathways simultaneously. Methods: A narrative review of peer-reviewed literature was conducted using PubMed. Searches combined the following MeSH terms: neuroinflammation, oxidative stress, retinal neurodegeneration, microglia, Müller glia, mitochondrial dysfunction, glaucoma, age-related macular degeneration, diabetic retinopathy, and Alzheimer’s disease. Priority was given to original research, systematic reviews, and high-impact publications from 2000 through 2025. However, seminal foundational works were included regardless of publication date. Studies were selected based on relevance to glial activation, mitochondrial dysfunction, reactive oxygen and nitrogen species, and disease-specific neuronal outcomes. Results: Across all four diseases, persistent microglial and Müller glial activation, mitochondrial electron transport chain dysfunction, and excess reactive oxygen species (ROS) and reactive nitrogen species (RNS) production form a self-amplifying feed-forward loop that accelerates neuronal injury. In glaucoma, these mechanisms drive intraocular pressure-independent retinal ganglion cell loss. In AMD and DR, lipid dysregulation, complement activation, and chronic hyperglycemia sustain oxidative-inflammatory injury to the retinal pigment epithelium, photoreceptors, and neurovasculature. In AD, retinal amyloid deposition and oxidative stress mirror cortical pathology, positioning the retina as a noninvasive biomarker site. Conclusions: Neuroinflammation and oxidative stress constitute unifying upstream mechanisms across major vision-threatening neurodegenerative diseases. Combination therapeutic strategies that simultaneously modulate glial activation and restore redox homeostasis may offer superior neuroprotective efficacy compared to approaches targeting isolated downstream mediators.

## 1. Introduction

Neurodegeneration of the visual system ranks among the most consequential and least reversible causes of disability in aging populations worldwide [1]. Glaucoma affects over 76 million people globally and is the leading cause of irreversible blindness. Age-related macular degeneration (AMD) is projected to affect approximately 288 million people by 2040. Diabetic retinopathy threatens vision in approximately one-third of individuals with diabetes. and Alzheimer’s disease, which increasingly manifests with retinal pathology, affects over 55 million people globally with no treatment yet proven to halt its underlying neurodegeneration [2,3,4,5]. Despite decades of research, current therapeutic strategies across all four conditions remain largely palliative, and do not durably halt the underlying neuronal loss that drives irreversible vision and cognitive decline [6,7].

A critical reason for this therapeutic limitation may be conceptual. These diseases have traditionally been studied in isolation, each assigned to its own clinical subspecialty and its own mechanistic narrative. Yet converging evidence from molecular biology, neuroimaging, and translational research increasingly reveals that glaucoma, AMD, DR, and AD share a common pathological architecture with chronic glial activation, mitochondrial dysfunction, excess reactive oxygen species (ROS) and reactive nitrogen species (RNS) production, and progressive neuronal loss driven by a mutually amplifying feed-forward loop between neuroinflammation and oxidative stress [8,9]. In each disease, persistent microglial and Müller glial activation generates an oxidative stress that further activates inflammatory signaling. This cycle operates upstream of the downstream mediators currently targeted by approved therapies and continues to drive neurodegeneration even when downstream targets are suppressed [7].

The retina occupies a unique position in this framework. As a direct extension of the central nervous system, it is subject to the same inflammatory and redox stresses that drive cortical and hippocampal neurodegeneration in AD, yet it remains optically accessible for noninvasive imaging and intervention in ways the brain does not [10]. Retinal thinning, microvascular abnormalities, and amyloid accumulation have been correlated with cognitive impairment and cortical atrophy, highlighting the potential of the retina as a biomarker site for early diagnosis, disease monitoring, and therapeutic assessment in AD and other CNS disorders [11,12].

Aging profoundly modulates both oxidative resilience and immune reactivity, lowering the threshold for chronic glial activation and mitochondrial dysfunction. The age-associated declines in mitophagy, antioxidant enzyme expression, and blood–retinal barrier integrity may prime retinal tissue for degeneration even in the absence of overt disease triggers. Emerging evidence also suggests sex-dependent differences in glial inflammatory responses and oxidative defenses, which may contribute to disparities in disease prevalence and progression. Incorporating age- and sex-aware frameworks will be essential for refining therapeutic strategies and clinical trial design.

This review synthesizes current evidence linking neuroinflammation and oxidative stress as interconnected, upstream drivers of neurodegeneration in glaucoma, AMD, DR, and AD. By framing these disorders within a shared mechanistic network, we outline the rationale for therapeutic strategies aimed at modulating glial reactivity and restoring redox balance. Ultimately, achieving durable neuroprotection will require moving beyond isolated downstream targets toward interventions that interrupt the inflammatory–oxidative axis at its origin.

## 2. Methods

A narrative review of peer-reviewed literature was conducted using PubMed. Searches combined the following MeSH terms: neuroinflammation, oxidative stress, retinal neurodegeneration, microglia, Müller glia, mitochondrial dysfunction, glaucoma, age-related macular degeneration, diabetic retinopathy, and Alzheimer’s disease. Priority was given to original research articles, systematic reviews, and high-impact publications from 2000 through 2025. However, seminal foundational works were included regardless of publication date. Studies were included if they examined glial activation, mitochondrial dysfunction, reactive oxygen and nitrogen species production, or disease-specific neuronal outcomes in the context of at least one of the four diseases under review. Studies were excluded if they were not published in peer-reviewed journals, not available in English, or focused exclusively on non-visual or non-CNS pathology without mechanistic relevance to the inflammatory–oxidative axis. Approximately 89 publications met these criteria and informed the synthesis presented here. As a narrative review, this study does not employ formal systematic methods such as PRISMA screening or meta-analytic pooling. This approach was chosen because the scope of the review spans four distinct disease contexts united by shared upstream mechanisms.

## 3. Neuroinflammation in Retinal and Central Degeneration

Neuroinflammation is a defining feature of both CNS and retinal degenerative diseases [13,14]. In the brain, microglia are resident innate immune cells that maintain tissue homeostasis under physiological conditions. In disease states, sustained microglial activation drives production of proinflammatory cytokines, chemokines, complement components, and ROS/RNS. These mediators perturb synaptic function by altering neurotransmitter receptor trafficking, promoting aberrant complement-dependent synaptic pruning, and inducing oxidative damage to synaptic proteins [15,16]. Axonal transport is likewise compromised as microtubules and motor proteins are damaged, disrupting anterograde and retrograde trafficking vital for neuronal maintenance [17,18]. Proinflammatory cytokines and ROS/RNS also promote neuronal apoptosis by activating death receptor signaling that triggers mitochondrial outer membrane permeabilization and caspase-dependent cell death [19,20].

Similar immune responses occur in the retina, where retinal microglia and Müller glia orchestrate inflammatory signaling [21]. Under pathological conditions such as glaucoma, AMD, and DR, retinal microglia migrate to injured sites and adopt activated phenotypes that amplify local inflammation [22,23]. Activated Müller glia further influence disease by altering metabolic support, glutamate clearance, and cytokine release. Notably, chronic glial activation often precedes overt neuronal loss, implicating immune dysregulation in disease initiation [1,24].

Dysfunction of the blood–retinal barrier (BRB) parallels blood–brain barrier breakdown in CNS disorders. Increased vascular permeability permits infiltration of peripheral immune cells and exposes neural tissue to systemic inflammatory mediators, highlighting the contribution of systemic inflammation and metabolic disease to retinal neurodegeneration [25,26].

Recent single-cell transcriptomic and spatial profiling studies reveal that neither retinal nor CNS glia exist as uniform populations but instead adopt disease- and context-specific activation states that distinctly influence neurodegenerative outcomes. Microglia transition from homeostatic phenotypes toward pro-inflammatory, neurotoxic, or alternatively neuroprotective states characterized by differential expression of complement genes, cytokines, lipid metabolism pathways, and antioxidant enzymes. Similarly, Müller glia exhibit reactive subtypes ranging from metabolically supportive responses to maladaptive gliosis that exacerbates inflammation and oxidative stress. These findings suggest that therapeutic strategies must move beyond global glial suppression toward selective reprogramming of pathogenic glial states, preserving beneficial immune surveillance while limiting chronic neurotoxicity.

## 4. Oxidative Stress and Mitochondrial Dysfunction in Vision-Threatening Disease

Oxidative stress arises when the production of ROS/RNS exceeds endogenous antioxidant capacity [23,27]. Neurons and photoreceptors are uniquely vulnerable because of high oxygen consumption, abundant polyunsaturated lipids, and limited regenerative capacity. In the retina, chronic light exposure and intense mitochondrial activity further exacerbate this oxidative stress.

Mitochondrial dysfunction is a central driver of oxidative stress in both retinal and cerebral neurodegeneration. Impaired electron transport chain activity, altered mitochondrial dynamics, and defective mitophagy reduce ATP production and increase reactive species generation. In retinal ganglion cells (RGCs), mitochondrial dysfunction is strongly implicated in glaucomatous degeneration, while in photoreceptors and retinal pigment epithelium (RPE), it plays a critical role in AMD pathogenesis [28,29]. Beyond direct macromolecular damage, oxidative stress alters redox-sensitive signaling pathways that regulate metabolism and cell survival, which promotes maladaptive gene expression and reinforces chronic inflammatory activation [30].

Immunometabolism reprogramming has emerged as a critical intermediary linking oxidative stress and chronic neuroinflammation. Activated microglia and Müller glia undergo metabolic shifts toward glycolysis and altered lipid utilization, increasing reactive oxygen species production while sustaining inflammatory gene expression. In AMD, oxidized lipids within drusen not only serve as markers of oxidative injury but also actively signal through pattern recognition receptors and complement pathways to perpetuate inflammation. Similar lipid-driven immune activation has been described in glaucomatous and diabetic retinas, suggesting that dysregulated lipid metabolism represents a shared, targetable node within the inflammatory–oxidative axis.

## 5. Bidirectional Communication Between Neuroinflammation and Oxidative Stress

Neuroinflammation and oxidative stress form an amplifying pathological network [9]. Activated glial cells generate reactive species as part of the inflammatory response, while oxidative stress activates intracellular cascades that enhance inflammatory gene transcription [8]. This feed-forward loop sustains tissue injury and accelerates neuronal dysfunction.

In retinal disease, oxidative stress arising from metabolic dysregulation or light exposure activates inflammatory pathways in microglia and Müller glia [31]. Conversely, inflammatory mediators increase oxidative stress by impairing mitochondrial function and antioxidant capacity. During persistent inflammation, ROS production can outpace mitochondrial enzymes such as SOD2, PRDX3, and GPX4 that normally limit oxidative injury [32]. This convergence helps explain why interventions that target isolated downstream mediators often show limited durability in halting disease progression [33].

**Figure 1:**
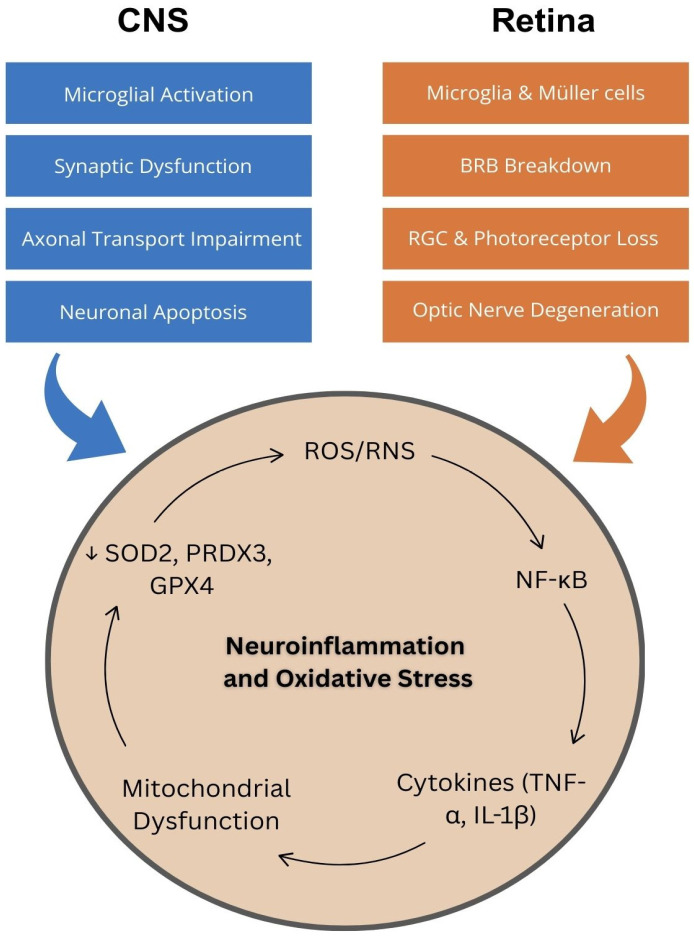
Convergent Pathological Mechanisms of Neuroinflammation and Oxidative Stress in CNS and Retinal Neurodegeneration. Schematic illustrating the shared neuroinflammatory and oxidative stress pathways underlying CNS and retinal neurodegeneration. Blue boxes (left) represent hallmark features of CNS pathology, including microglial activation, synaptic dysfunction, axonal transport impairment, and neuronal apoptosis. Orange boxes (right) depict analogous retinal manifestations, including activation of microglia and Müller glia, blood–retinal barrier breakdown, retinal ganglion cell and photoreceptor loss, and optic nerve degeneration. The central circle illustrates the self-amplifying feed-forward loop between reactive oxygen species and reactive nitrogen species (ROS/RNS), NF-κB activation, proinflammatory cytokine release (TNF-α, IL-1β), mitochondrial dysfunction, and depletion of antioxidant enzymes (SOD2, PRDX3, GPX4). Both CNS and retinal pathology converge on this shared mechanistic cycle, emphasizing the rationale for therapeutic strategies targeting root mechanisms of neurodegeneration. Abbreviations: CNS, central nervous system; BRB, blood–retinal barrier; RGC, retinal ganglion cell; ROS/RNS, reactive oxygen and nitrogen species; SOD2, superoxide dismutase 2. ↓ indicates downregulation.

The transcription factor NF-κB mediates this bidirectional loop. Because it is activated by both inflammatory cytokines and ROS-mediated oxidative signals, NF-κB drives transcription of TNF-α, IL-1β, iNOS, and NADPH oxidase subunits. These mediators sustain glial activation and amplify superoxide production [8,34]. Simultaneously, oxidative modification of IκB, the endogenous NF-κB inhibitor, prevents its reassociation with the NF-κB complex, locking the pathway in a constitutively active state even after the initiating stimulus resolves [8]. The Nrf2 pathway operates in functional opposition: under homeostatic conditions, Nrf2 transcriptionally upregulates antioxidant enzymes including HO-1, NQO1, and the glutamate-cysteine ligase subunits that replenish glutathione, collectively dampening both oxidative stress and NF-κB-driven inflammation [35]. In degenerating retinal and CNS tissue, however, chronic oxidative stress promotes Nrf2 sequestration and degradation, dismantling this endogenous brake precisely when it is most needed [35,36]. The result is a molecular environment in which the pro-inflammatory and pro-oxidant arms reinforce one another at the transcriptional level, helping to explain why neurodegeneration persists and accelerates independent of the upstream stressor that originally initiated it (Figure 1).

## 6. Disease-Specific Contexts

### 6.1. Glaucoma

Glaucoma is increasingly recognized as a complex neurodegenerative disease of the visual system, rather than solely a disorder of elevated intraocular pressure (IOP), characterized by progressive RGC loss and optic nerve degeneration [37]. Although elevated IOP remains the primary risk factor and therapeutic focus, many patients continue to progress despite adequate pressure control, implicating additional mechanisms that operate independently of IOP [7].

Neuroinflammation contributes centrally to pressure-independent RGC death. In glaucomatous eyes, microglia and astrocytes adopt activated phenotypes and secrete proinflammatory cytokines, including TNF-α and IL-1β, that induce local neurotoxicity and compromise axonal structure. Glial activation engages redox-sensitive pathways that amplify inflammation and diminish neuroprotective support, particularly when antioxidant defenses are weakened [38,39]. Experimental models show that inhibiting glial NF-κB signaling mitigates secondary neuronal injury and reduces RGC loss, underscoring the role of immune dysregulation in glaucomatous neurodegeneration [34].

Oxidative stress is tightly intertwined with inflammation in glaucoma. RGCs and optic nerve tissues are vulnerable to ROS accumulation when antioxidant homeostasis is disrupted. Excess ROS directly damages lipids, proteins, and DNA and acts as secondary messengers that further activate inflammatory signaling. Oxidative modifications of retinal proteins and elevated markers of oxidative stress have been documented in glaucomatous tissues, reinforcing the role of redox imbalance in disease pathogenesis [40]. Antioxidants and immunomodulatory interventions reduce inflammatory cytokine production and ROS damage in preclinical models, suggesting opportunities for neuroprotection beyond IOP lowering [41].

Nonetheless, translation to clinical benefit has proven challenging with neuroprotective agents that succeeded in animal models largely failing to demonstrate efficacy in randomized trials. This likely reflects the complexity of human glaucomatous neurodegeneration, the limitations of IOP-controlled animal models, and the challenge of delivering sufficient concentrations to the optic nerve head over the required therapeutic window [37,42].

### 6.2. Age-Related Macular Degeneration (AMD)

AMD is a multifactorial disease emerging from interactions among oxidative stress, lipid metabolic dysregulation, chronic inflammation, and complement pathway activation. It is the leading cause of visual impairment and blindness in older adults in the United States [43]. RPE and photoreceptor cells are highly metabolically active and light-exposed, rendering them susceptible to ROS-mediated damage. Accumulating oxidative injury to proteins and DNA contributes to cellular dysfunction and death [44].

A hallmark of early and intermediate AMD is the formation of subretinal drusen that contain esterified and unesterified cholesterol, apolipoproteins, and oxidized lipid products. Drusen reflect disrupted lipid handling: failure of the RPE to recycle lipids from photoreceptor outer segments and to sustain reverse cholesterol transport prompts secretion of ApoB-containing lipoproteins into Bruch’s membrane, initiating lipid accumulation reminiscent of atherosclerotic plaques [45]. Chronic inflammation in the retina and choroid, fueled by resident and infiltrating immune cells, cytokines, and complement, drives lesion progression [46]. Variants in complement genes such as CFH, C3, and CFB reduce regulatory control, increase AMD risk, and promote persistent macular inflammation [47]. The relationship between oxidative damage and complement-mediated inflammation in AMD remains unclear and likely varies across disease stages and genetic profiles. This hinders the rational sequencing of therapeutic targets [46,47]. Trials targeting complement alone have produced modest and variable effects on atrophy progression, suggesting that isolated complement inhibition may be insufficient without concurrent antioxidant support [48,49]. This observation reinforces the case for combination strategies addressing multiple nodes of the inflammatory-oxidative loop simultaneously.

### 6.3. Diabetic Retinopathy (DR)

Although classically defined as a microvascular complication of diabetes, DR also exhibits early neurodegenerative features. Functional and structural abnormalities in retinal neurons, glia, and the neurovasculature precede overt vascular pathology [50,51]. Chronic hyperglycemia sustains oxidative stress through the polyol pathway, protein kinase C signaling, and advanced glycation end-product (AGE) formation, while mitochondrial electron transport defects further increase ROS and overwhelm endogenous antioxidant defenses [29,52].

Oxidative stress is closely coupled to low-grade chronic inflammation in DR. Hyperglycemia and ROS activate retinal microglia and Müller glia, driving the release of cytokines and chemokines that recruit peripheral immune cells [31]. This inflammatory milieu disrupts neurotrophic support and contributes to early RGC apoptosis [53]. Concurrently, inflammatory signaling accelerates BRB breakdown and vascular permeability, linking neuronal and vascular injury. ROS–inflammation feedback loops exacerbate mitochondrial dysfunction, neurovascular uncoupling, and progressive retinal degeneration [54]. Importantly, the temporal dissociation between glycemic control and neurodegeneration in DR is notable: intensive glucose management reduces the risk of incident retinopathy but has a limited effect on halting established neurodegeneration, suggesting that once the inflammatory-oxidative cascade is initiated, it becomes partially independent of the metabolic trigger [50,55]. This has direct implications for treatment windows and argues for early neuroprotective intervention before the feedback loop becomes self-sustaining.

### 6.4. Alzheimer’s Disease (AD) and Retinal Neurodegeneration

Alzheimer’s disease (AD) shares mechanistic parallels with retinal neurodegenerative disorders. Retinal manifestations, including amyloid-β deposition, microglial and astrocytic activation, synaptic dysfunction, and heightened oxidative stress, mirror brain pathology and have been increasingly recognized in postmortem retinal tissue and in vivo imaging studies [12]. As an accessible extension of the CNS, the retina offers a window into early neurodegenerative changes that may precede clinical cognitive symptoms.

Retinal amyloid accumulation is associated with local inflammatory responses characterized by microglial activation and proinflammatory cytokine release that disrupt neural homeostasis. Activated microglia in the AD retina adopt proinflammatory phenotypes that amplify oxidative stress and compromise synaptic integrity through mechanisms directly analogous to those operating in the hippocampus and cortex [15,56]. Excess ROS generated through mitochondrial dysfunction contributes to lipid peroxidation, DNA damage, and protein oxidation, reinforcing a cycle that mimics cortical and hippocampal pathology [11,57].

The retina’s potential as a biomarker site for AD is supported by retinal nerve fiber layer thinning and the association between ganglion cell complex metrics, cortical atrophy, and cognitive decline. Retinal amyloid imaging has demonstrated correlations with PET-confirmed cerebral amyloid burden, while alterations in retinal vascular geometry reflect cerebrovascular changes linked to AD risk [11,58,59]. Emerging artificial intelligence approaches are increasingly being applied to retinal imaging data to enhance the sensitivity and specificity of these associations. Machine learning algorithms trained on optical coherence tomography (OCT) and fundus photography have shown early promise in distinguishing AD-related retinal changes from those attributable to aging, glaucoma, or DR, and in correlating quantitative retinal features with amyloid burden and cognitive status. These AI-assisted approaches may ultimately enable non-invasive, population-scale screening for preclinical AD, though their prospective validation across diverse cohorts and standardization across imaging platforms remain essential prerequisites for clinical deployment [60].

On the therapeutic side, amyloid-targeting strategies are also evolving beyond general antioxidant or anti-inflammatory approaches toward more specific interventions. Anti-amyloid monoclonal antibodies, including lecanemab and donanemab, have recently demonstrated slowing of cognitive decline in early AD in randomized trials, establishing proof-of-concept that amyloid clearance can produce clinical benefit [61,62]. Whether these systemic therapies reduce retinal amyloid burden in parallel with cerebral clearance, and whether retinal imaging can serve as a surrogate endpoint for tracking treatment response, are open questions with significant translational relevance.

Small molecules designed to inhibit amyloid-β aggregation or promote its clearance via the glymphatic or retinal lymphatic systems represent additional investigational avenues, though their ocular pharmacokinetics and target engagement in the retina have not yet been characterized [63,64]. While these findings are promising, important caveats remain. Correlation between retinal and brain pathology is not uniform across AD subtypes, longitudinal data linking retinal changes to cognitive trajectories remain limited, and imaging protocols have yet to be standardized or validated across diverse populations [65]. Most critically, retinal neurodegeneration is not specific to AD. It occurs in glaucoma and DR as well, and distinguishing AD-related changes from those driven by other degenerative processes remains an unsolved challenge for clinical translation. The convergence of amyloid pathology, neuroinflammation, and oxidative stress in both the retina and brain nonetheless supports the retina as a noninvasive site for both biomarker detection and therapeutic assessment in CNS neurodegeneration, and justifies targeting shared upstream pathways in parallel with more disease-specific interventions. The convergent pathological mechanisms discussed across all four diseases are summarized in Table 1.

## 7. Therapeutic Implications for Vision Science

Recognizing neuroinflammation and oxidative stress as central, interconnected initiating mechanisms of retinal neurodegeneration argues for combination therapies that modulate glial activation and bolster antioxidant defenses. Precise approaches should seek to shift glial states toward neuroprotection and metabolic support, paired with interventions that restore mitochondrial function, improve redox buffering, and dampen maladaptive inflammatory signaling [28,29,66]. Such multimodal strategies may better interrupt the inflammatory–oxidative feedback loop that accelerates neurodegeneration.

Several therapeutic strategies targeting these converging pathways have demonstrated preclinical or early clinical promise. Antioxidant-based interventions represent the most clinically advanced approach. N-acetylcysteine (NAC), a glutathione precursor and direct ROS scavenger, reduces oxidative damage and attenuates microglial activation in models of glaucoma and DR [67,68]. The AREDS and AREDS2 formulations remain the only interventions with demonstrated efficacy in slowing AMD progression in patients with intermediate or advanced unilateral disease, which validates the antioxidant hypothesis at the clinical level. However, their benefit is largely preventive rather than restorative, and they do not address the inflammatory component directly [44,69].

Nrf2 pathway activation offers another antioxidant strategy. Nrf2 is the master transcriptional regulator of the cellular antioxidant response, governing expression of heme oxygenase-1 (HO-1), NQO1, and glutamate-cysteine ligase among others [35]. Pharmacological Nrf2 activators, including sulforaphane and dimethyl fumarate, have demonstrated neuroprotective effects in rodent models of glaucoma and AMD by reducing ROS accumulation, suppressing NF-κB-mediated inflammatory gene expression, and preserving mitochondrial membrane potential [36,70]. Nrf2 activation simultaneously engages antioxidant and anti-inflammatory transcriptional programs, making it a biologically rational node for combination therapy [35]. Clinical translation to retinal indications remains at an early stage, and the narrow therapeutic window of some activators requires careful dose optimization [71].

Glial-targeted immunomodulation offers a different strategy that reduces inflammation. Minocycline, a tetracycline-class antibiotic with microglial inhibitory properties, reduces proinflammatory cytokine release and ROS production in models of glaucoma and DR [72,73]. NF-κB inhibition has shown efficacy in experimental glaucoma, with transgenic astroglial NF-κB suppression preserving RGC survival and attenuating optic nerve degeneration [34]. Complement inhibition has reached clinical validation for retinal neurodegeneration. Intravitreal avacincaptad pegol, a C5 inhibitor, demonstrated significant slowing of geographic atrophy lesion growth and received FDA approval in 2023, representing a landmark demonstration that targeting inflammatory upstream mediators can produce meaningful clinical benefit [74]. However, these approvals address a late stage of AMD rather than the neuroinflammatory process itself, and efficacy on photoreceptor and neuronal preservation remains incompletely characterized [75].

Mitochondria-targeted therapies address the organelle-level source of oxidative stress. MitoQ and SkQ1 are mitochondria-targeted antioxidants that accumulate selectively within the mitochondrial matrix, neutralizing superoxide at its site of production [76]. Preclinical data in models of AMD and glaucoma show the preservation of RPE and RGC viability alongside reduced inflammatory activation, respectively [77,78,79]. NAD+ precursors such as nicotinamide riboside (NR) and nicotinamide mononucleotide (NMN) support mitochondrial bioenergetics by replenishing NAD+ pools depleted by oxidative stress in glaucoma [80,81]. This strategy is relevant to RGC survival in glaucoma, where mitochondrial fragmentation and energy failure precede axonal degeneration [28,29].

Translating combination therapeutic strategies from preclinical models to clinical practice introduces delivery challenges that are distinct from those associated with monotherapy. The ocular environment imposes significant pharmacokinetic barriers: tear turnover, nasolacrimal drainage, and the corneal epithelial barrier collectively limit the bioavailability of topically applied agents to less than 5% of the administered dose, and this limitation is compounded when two agents with differing physicochemical properties must reach the same target tissue simultaneously [82]. For posterior segment targets such as the RPE, photoreceptors, optic nerve head, and inner limiting membrane, intravitreal delivery circumvents anterior barriers. In doing so, it introduces distinct challenges such as restricted diffusion through the vitreous, variable retinal penetration depending on molecular weight and charge, and the burden of repeated injections required to maintain therapeutic concentrations over the chronic disease course [83]. When two agents are co-administered intravitreally, differences in molecular weight, charge, and protein binding mean that the two compounds are unlikely to maintain comparable therapeutic concentrations at the target tissue over time. This complicates the rational design of dosing intervals for combination regimens since their inherent differences determine their vitreous distribution and elimination [84]. Systemic delivery, while appealing for its non-invasive route, has repeatedly failed to achieve sufficient intraocular concentrations to overcome localized oxidative–inflammatory loops, as demonstrated by the limited efficacy of oral antioxidant supplementation beyond the AREDS formulation in advanced disease [85]. Sustained-release platforms, such as biodegradable intravitreal implants, nanoparticle carriers, and hydrogel depots, offer a potential path forward by enabling controlled co-release of antioxidant and immunomodulatory agents at the target site over extended periods [86]. However, these platforms remain largely investigational for neuroinflammatory indications, and their long-term safety profiles in the context of chronic neurodegenerative disease have not yet been established. Addressing these delivery barriers is a prerequisite for realizing the clinical potential of combination approaches targeting the inflammatory–oxidative axis.

Antioxidant supplementation beyond the AREDS formulation has not demonstrated consistent efficacy in advanced AMD or glaucoma, demonstrating that systemic delivery is insufficient to overcome localized oxidative–inflammatory loops [85,87]. Single-pathway targeting has repeatedly shown limited durability since lowering the IOP alone does not stop glaucoma progression in a substantial minority of patients and VEGF suppression in DR requires indefinite re-treatment while not addressing the underlying neuronal loss [6,88,89]. The pattern of partial responses to monotherapy across all four diseases reviewed here points consistently toward combination strategies that address the inflammatory–oxidative axis in parallel.

## 8. Limitations

Several limitations should be considered when interpreting the conclusions of this review. First, the cross-disease framework presented here, while mechanistically justified, risks overgeneralizing the shared inflammatory–oxidative axis in ways that obscure clinically important disease-specific differences. The relative contributions of glial activation and mitochondrial dysfunction differ substantially across glaucoma, AMD, DR, and AD. In glaucoma, RGC vulnerability and optic nerve head biomechanics introduce disease-specific factors that are not captured by the shared feed-forward loop model. In AMD, the dominant role of the RPE and the strong influence of complement genetic variants, particularly CFH, C3, and CFB, introduce a genomic dimension that modulates both disease risk and therapeutic response in ways that vary across individuals and disease stages [47]. In DR, the temporal dissociation between glycemic control and established neurodegeneration highlights that the inflammatory–oxidative cascade, once initiated, acquires partial independence from its metabolic trigger [50,55]. In AD, the correlation between retinal and cortical pathology is not uniform across disease subtypes, and retinal neurodegeneration is not specific to AD, limiting the specificity of the retina as a biomarker site in the absence of disease-specific molecular signatures [65]. These distinctions mean that combination therapeutic strategies targeting the shared axis will likely require disease-specific adaptation in terms of target cell type, intervention timing, delivery route, and endpoint selection.

Second, most preclinical evidence reviewed here derives from rodent models, which imperfectly replicate the complexity of human neurodegenerative disease. The repeated failure of neuroprotective agents in clinical trials after promising animal model results, particularly in glaucoma, underscores the translational gap that remains a central challenge for the field [37,42].

Finally, the combination therapy rationale advanced here is largely inferential: direct evidence from randomized trials testing simultaneous glial modulation and antioxidant restoration in any of the four diseases under review is limited. The evidence base is composed primarily of mechanistic studies and single-agent trials whose results are synthesized to support a logical, but yet unproven, combination approach.

## 9. Future Directions and Outstanding Questions

### 9.1. Precision Modulation of Glial States

Future therapies must aim to selectively suppress neurotoxic glial phenotypes while preserving protective immune functions. Advances in single-cell and spatial transcriptomics offer an unprecedented opportunity to identify disease-specific glial states and their regulatory checkpoints. Therapeutic strategies targeting metabolic or transcriptional regulators that govern glial state transitions may enable more precise immunomodulation than global anti-inflammatory approaches.

### 9.2. Combination and Sequencing Therapies

The limited durability of monotherapies across glaucoma, AMD, DR, and AD argues for rational combination strategies that address multiple nodes within the inflammatory–oxidative network. An unresolved challenge is determining optimal therapeutic sequencing, whether early antioxidant or mitochondrial support should precede immunomodulation, or whether simultaneous intervention yields superior neuroprotection. Preclinical studies explicitly comparing these strategies are urgently needed.

### 9.3. Retina-Based Biomarkers and Treatment Windows

Harnessing the retina as a biomarker site for CNS neurodegeneration requires longitudinal studies linking retinal inflammatory and oxidative signatures to cognitive decline and therapeutic response. Developing disease-specific retinal biomarkers that distinguish AD from other retinal neurodegenerations remains a critical barrier. Standardization of imaging protocols and integration with systemic biomarkers will be essential for clinical translation.

### 9.4. Translational Challenges and Delivery

Finally, effective delivery of antioxidant and immunomodulatory therapies to retinal and optic nerve tissues remains a limiting factor. Emerging approaches including sustained-release intravitreal formulations, nanoparticle-based delivery, and gene-based modulation of antioxidant pathways warrant systematic evaluation.

## 10. Conclusions

Neuroinflammation and oxidative stress are unifying upstream mechanisms in neurodegenerative diseases of the brain and visual system. Glaucoma, AMD, DR, and Alzheimer’s disease share common pathological pathways: sustained glial activation, mitochondrial dysfunction, ROS/RNS excess, and barrier breakdown. These factors culminate in progressive neuronal dysfunction and loss through a self-sustaining loop. Framing these disorders within shared pathology points away from isolated downstream targets and directs therapeutic strategies toward interrupting the degenerative cascade at its source. Durable neuroprotection will likely require combination approaches that simultaneously modulate glial state, restore redox homeostasis, and support mitochondrial bioenergetics. The retina, as both a vulnerable target of these processes and an optically accessible extension of the CNS, offers a site where mechanisms can be studied and therapeutically targeted.

## Figures and Tables

**Table 1:** Convergent Pathological Mechanisms Across Neurodegenerative Diseases of the Visual System and Brain. Abbreviations: IOP, intraocular pressure; RGC, retinal ganglion cell; BBB, blood–brain barrier; ROS, reactive oxygen species; RPE, retinal pigment epithelium; PKC, protein kinase C; AGE, advanced glycation end-products; CFH, complement factor H; AMD, age-related macular degeneration; TNF-α, tumor necrosis factor-alpha; IL-1β, Interleukin-1β; IL-6, Interleukin-6; NF-κB, nuclear factor kappa-light-chain-enhancer of activated B cells.

	Glaucoma	AMD	Diabetic Retinopathy	Alzheimer’s Disease
Upstream Stressor	– Elevated IOP – Axonal transport impairment	– Aging – Light exposure – Lipid metabolic dysregulation	– Chronic hyperglycemia	– Amyloid plaques – Tau pathology
Inflammatory Drivers	– Microglial activation – Astrocyte reactivity – TNF-α/IL-1β signaling – NF-κB activation	– Complement activation (CFH, C3 variants) – Subretinal microglial accumulation	– Microglial activation – Cytokine release (TNF-α, IL-6) – Leukostasis	– Microglial activation – Proinflammatory cytokine release
Oxidative/ Mitochondrial	– Mitochondrial fragmentation in RGCs – Decreased ATP production – ROS accumulation – Impaired antioxidant defense	– RPE mitochondrial dysfunction – Lipid peroxidation – Accumulation of oxidized lipoproteins	– Polyol pathway activation – PKC signaling – AGE formation – Mitochondrial superoxide overproduction	– Mitochondrial dysfunction – Lipid peroxidation – DNA + protein oxidation
Barrier/Vascular	– BRB dysfunction	– Bruch’s membrane thickening – Drusen formation	– BRB breakdown – Capillary dropout – Increased vascular permeability	– BBB disruption
Neuronal Outcomes	– RGC apoptosis – Optic nerve degeneration	– RPE dysfunction – Photoreceptor degeneration	– Early RGC apoptosis – Neurovascular uncoupling	– Synaptic loss – Cortical and hippocampal neurodegeneration – Retinal amyloid deposition

## Data Availability

Data are available within the article.
